# Supplementary material for: Exogenous L-arginine increases intestinal stem cell function through CD90+ stromal cells producing mTORC1-induced Wnt2b
Source: Commun Biol. 2020 Oct 23;3:611. doi: 10.1038/s42003-020-01347-9 (PMC7584578; doi:10.1038/s42003-020-01347-9)

Supplementary Information

Supplementary Figure 1

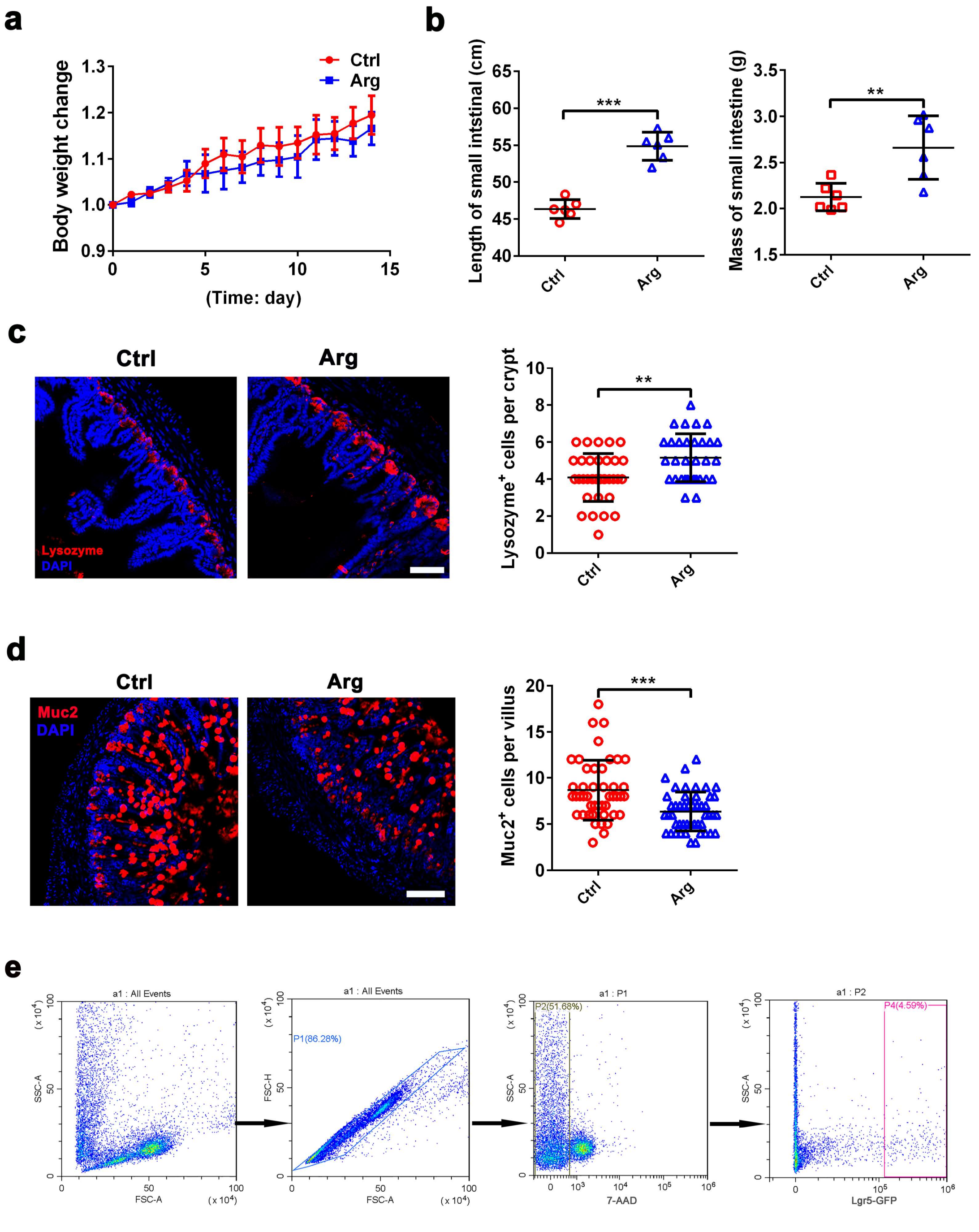

Supplementary Figure 1. Effects of L-arginine on body weight and SI development in mice, related to Figure 1

(A) Changes in body weight were monitored daily starting from day 1 to day 14 and presented relative to the initial body weight, n = 6. (B) The length and mass of small intestine were counted, n = 6. (C) Immunostaining of Lysozyme (red) and DAPI (blue) in small intestine. Scale bar, 50 μm. The number of $Lysozyme^+$ cell per crypt was counted, n = 3 mice per group. (D) Immunostaining of Muc2 (red) and DAPI (blue) in small intestine. Scale bar, 50 μm. The number of $Muc2^+$ cell per villus was counted, n = 3 mice per group. (E) The full gating strategy for the identification of the live $Lgr5^{hi}$ cells by flow cytometry. Data are the mean ± SD; comparisons performed with *t*-tests (two groups) or analysis of variance (ANOVA) (multiple groups). *$P < 0.05$, **$P < 0.01$, ***$P < 0.001$. Results are representative of two or three independent experiments.

Supplementary Figure 2

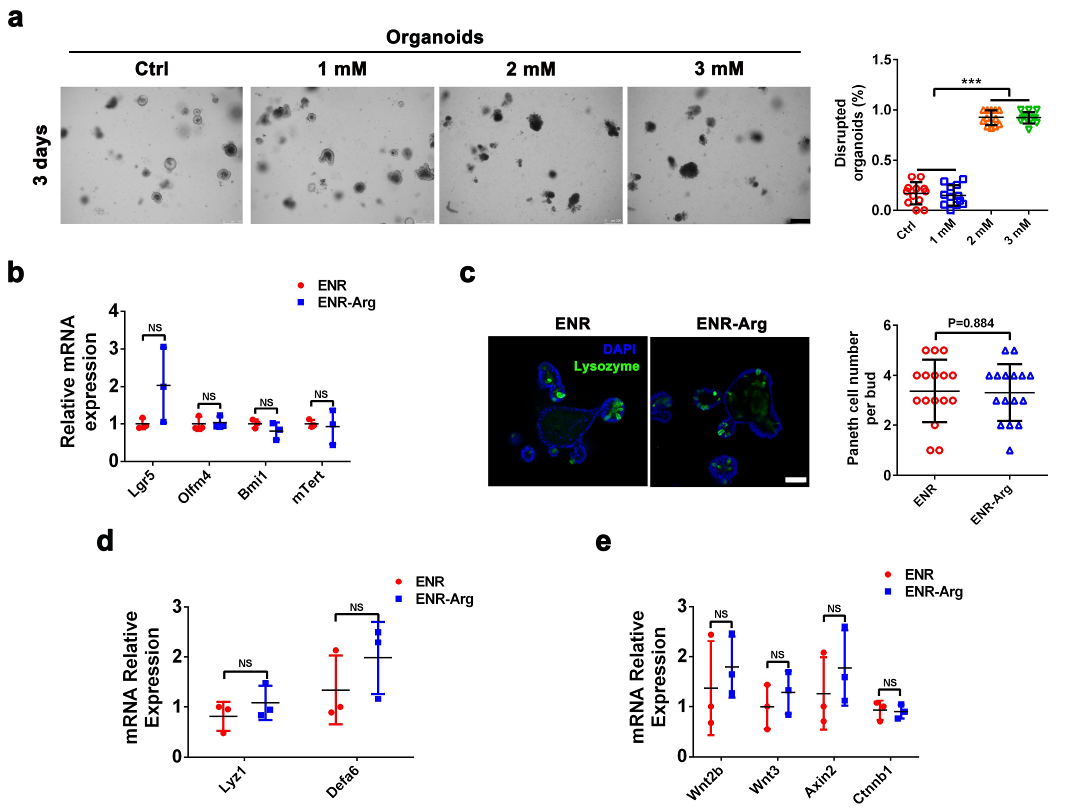

Supplementary Figure 2. The effects of L-arginine supplement on ISC function are not be mediated through Paneth cells niche, related to Figure 2 and 3

(a) The light microscope observation of SI organoids treated with different concentration of L-arginine (1 mM, 2mM, 3mM). Scale bar, 250 μm. The number of total organoids and disrupted organoids with altered morphology per well were counted, n = 12. (b) qPCR of relative mRNA expression of *Lgr5, Olfm4, Bmi1,* and *mTert* genes in SI organoids treated with 1 mM L-arginine or not. Expression show is relative to *GAPDH* gene, n = 3. (c) Immunostaining of Lysozyme (green) and DAPI (blue) in SI organoids. Scale bar, 50 μm. The number of Lysozyme^{+} cell per bud was counted, n = 16 buds per group. (d) qPCR of relative mRNA expression of *Lyz1* and *Defa6* genes in SI organoids treated with 1 mM L-arginine or not. Expression show is relative to *GAPDH* gene, n = 3. (e) qPCR of relative mRNA expression of *Wnt2b, Wnt3, Axin2,* and *Ctnnb1* genes in SI organoids treated with 1 mM L-arginine or not. Expression

show is relative to *GAPDH* gene, n = 3. Data are the mean ± SD; comparisons performed with *t*-tests (two groups) or analysis of variance (ANOVA) (multiple groups). $^{*}P < 0.05$, $^{**}P < 0.01$, $^{***}P < 0.001$. Results are representative of two or three independent experiments.

Supplementary Figure 3

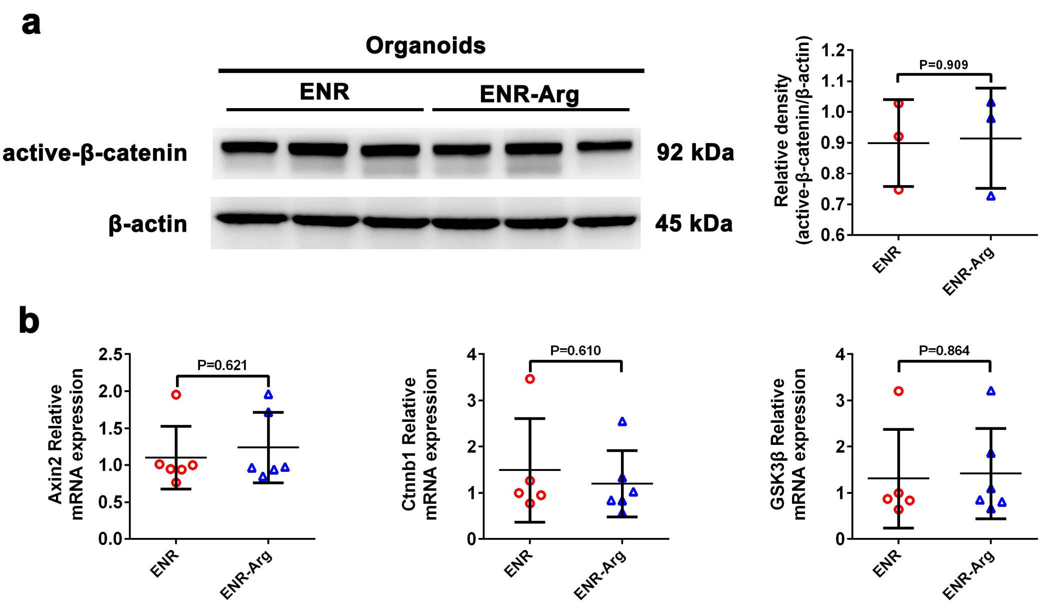

Supplementary Figure 3. L-arginine supplement failed to active Wnt/β-catenin pathway in SI organoids without CD90⁺ stromal cells, related to Figure 4

SI organoids were cultured with ENR-medium or ENR-medium supplemented 1 mM L-arginine for 72 h respectively. (a) Nuclear protein levels of GAPDH and active β-catenin were measured by western blotting assay in the SI organoids, n = 3. (b) qPCR of relative mRNA expression of *Ctnnb1*, *Axin2*, and *GSK3β* genes of the Wnt/β-catenin axis in SI organoids. Expression show is relative to *GAPDH* gene, n = 3. Data are the mean ± SD; comparisons performed with *t*-tests (two groups) or analysis of variance (ANOVA) (multiple groups). **P* < 0.05, ***P* < 0.01, ****P* < 0.001. Results are representative of two or three independent experiments.

Supplementary Figure 4

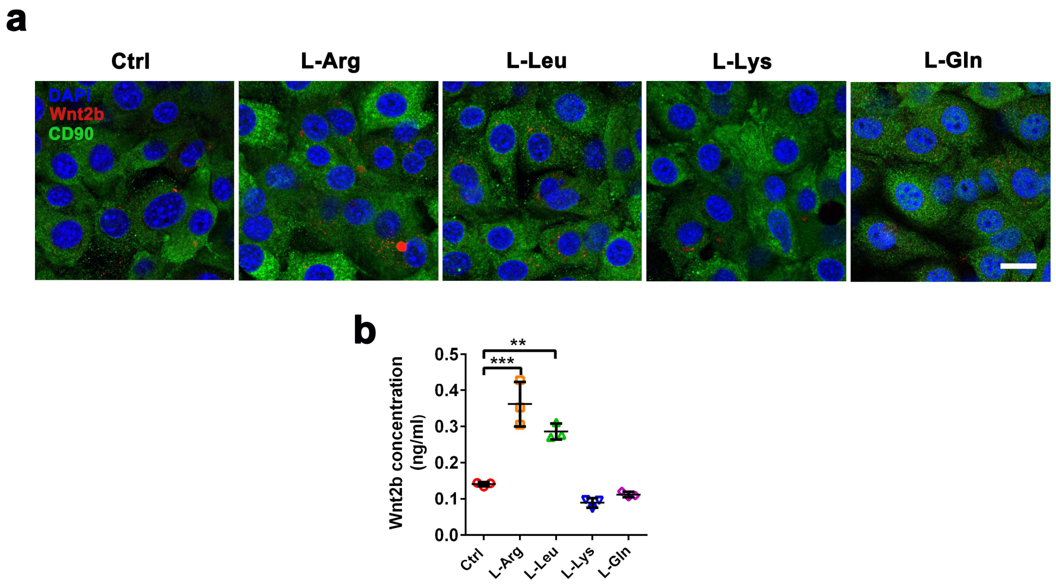

Supplementary Figure 4. The medium of CD90$^+$ stromal cells was supplemented with 1 mM different amino acids (L-arginine, L-leucine, L-lysine, L-glutamine), respectively**.** (A) Immunostaining of Wnt2b (red), CD90 (green) and DAPI (blue) in CD90$^+$ stromal cells. Scale bar, 5 µm. (B) The concentration of WNT2B in CD90$^+$ stromal cells culture supernatant was detected by ELISA, n = 3. Data are the mean ± SD; comparisons performed with *t*-tests (two groups) or analysis of variance (ANOVA) (multiple groups). $^*P < 0.05$, $^{**}P < 0.01$, $^{***}P < 0.001$. Results are representative of two or three independent experiments.

Supplementary Figure 5

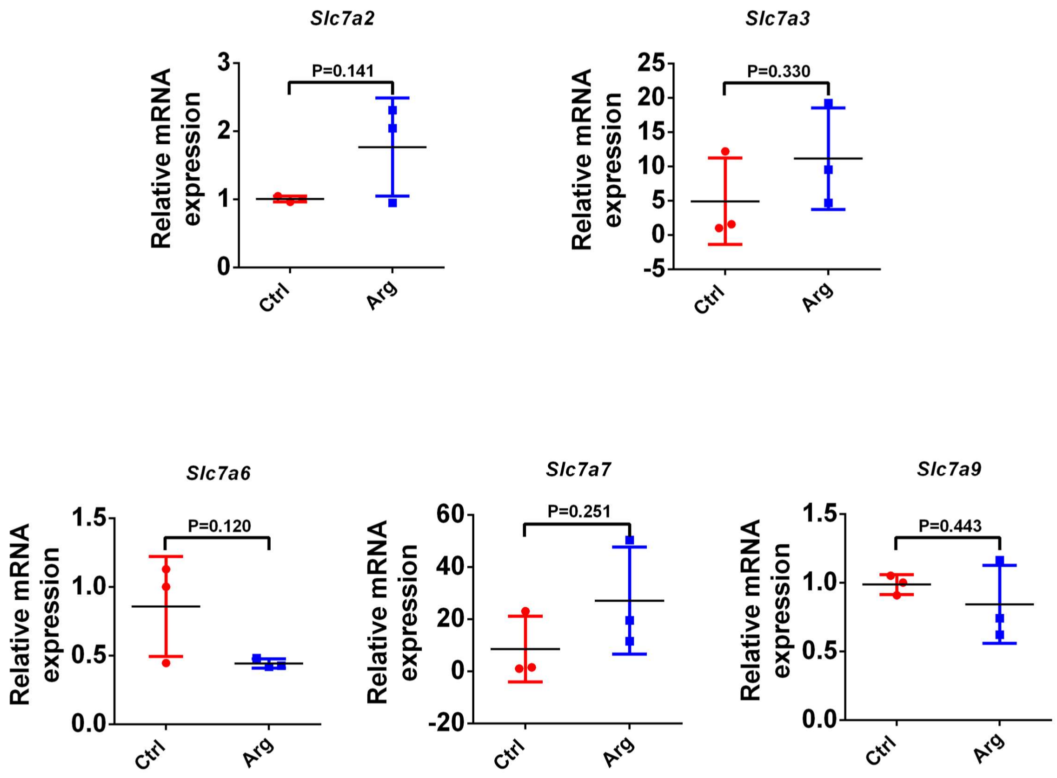

Supplementary Figure 5. Effect of L-arginine on the expression of amino acids transporter in CD90^{+} stromal cells, related to Figure 6

qPCR of relative mRNA expression of *Slc7a2*, *Slc7a3*, *Slc7a6*, *Slc7a7*, and *Slc7a9* genes in stromal cells. Expression show is relative to *GAPDH* gene, n = 3. Data are the mean ± SD; comparisons performed with *t*-tests (two groups) or analysis of variance (ANOVA) (multiple groups). *P < 0.05, **P < 0.01, ***P < 0.001. Results are representative of two or three independent experiments.

Supplementary Table 1. Primer sequences used for qRT-PCR.

Target genes	Primer sense (5'-3')	Primer antisense (5'-3')	Product size (bp)
mGSK3β	ACCGAGAACCACCTCCTTTG	TCACAGGGAGTGTCTGCTTG	165
mAxin2	TGACTCTCCTTCCAGATCCCA	TGCCCACACTAGGCTGACA	105
mCtnnb1	GTCAGCCACCAAGGTCGTTCATC	GTCGCTGCCATTGGAGGTCTTG	166
mLgr5	CCTACTCGAAGACTTACCCAGT	GCATTGGGGTGAATGATAGCA	165
mOlfm4	GTGCATGACAGAAAGGACGCT	CTGCTATACACAGGTTTCAGGAGC	175
mBmi1	ATCCCCACTTAATGTGTGTCCT	CTTGCTGGTCTCCAAGTAACG	116
mmTert	TCTACCGCACTTTGGTTGCC	CAGCACGTTTCTCTCGTTGC	160
mLyz1	GAGACCGAAGCACCGACTATG	CGGTTTTGACATTGTGTTCGC	214
mDefa6	CTAAAACTGAGGAGCAGCCAGG	CTTCCTTTGCAGCCTCTTGCTC	145
mWnt1	ATGAACCTTCACAACAACGAG	GGTTGCTGCCTCGGTTG	205
mWnt2b	CGTTCGTCTATGCTATCTCGTCAG	ACACCGTAATGGATGTTGTCACTAC	170
mWnt3	TGTGTCCAAGCTGCCTCTACT	TCCCCATCACTACATCCCAGC	165
mWnt3a	CACCACCGTCAGCAACAGCC	AGGAGCGTGTCACTGCGAAAG	214
mWnt5a	CTCCTTCGCCCAGGTTGTTATAG	TGTCTTCGCACCTTCTCCAATG	97
mWnt6	TGCCCGAGGCGCAAGACTG	ATTGCAAACACGAAAGCTGTCTCTC	130
mR-spondin1	GGGATCAAGGGCAAGAGACAG	CTGGCGGATGTCGTTCCTC	200
mGremlin 1	CTGGGGACCCTACTGCCAA	TTTGCACCAATCTCGCTTCAG	243
mGAPDH	GGCTGTATTCCCCTCCATCG	CCAGTTGGTAACAATGCCATGT	154

mSlc7a1	CTGCCTCAACACCTATGACCT	GAGAGCAGCAATCAAGAAGGAG	136
mSlc7a2	TCTATGTTCCCCTTACCCCGA	TGACTGCCTCTTACTCACTCTT	90
mSlc7a3	GGAGCTGGGTATGGGTGAGA	TGGATGGTCCTGCTTTATCTTTG	140
mSlc7a4	CCTTCTCAACACTTGCCTCAT	TGCCATAGCCAAAATACACGAC	101
mSlc7a6	GCCTGCGTATGTCTGCTGA	GCCCATGATAATGATGGCAATGA	117
mSlc7a7	AGCACCAAGTATGAAGTGGCT	ACACGCCATTAAGCAGGGAG	124
mSlc7a9	GAGGAGACGGAGAGAGGATGA	GAGGAGACGGAGAGAGGATGA	172

Original western blots in Figure 6C

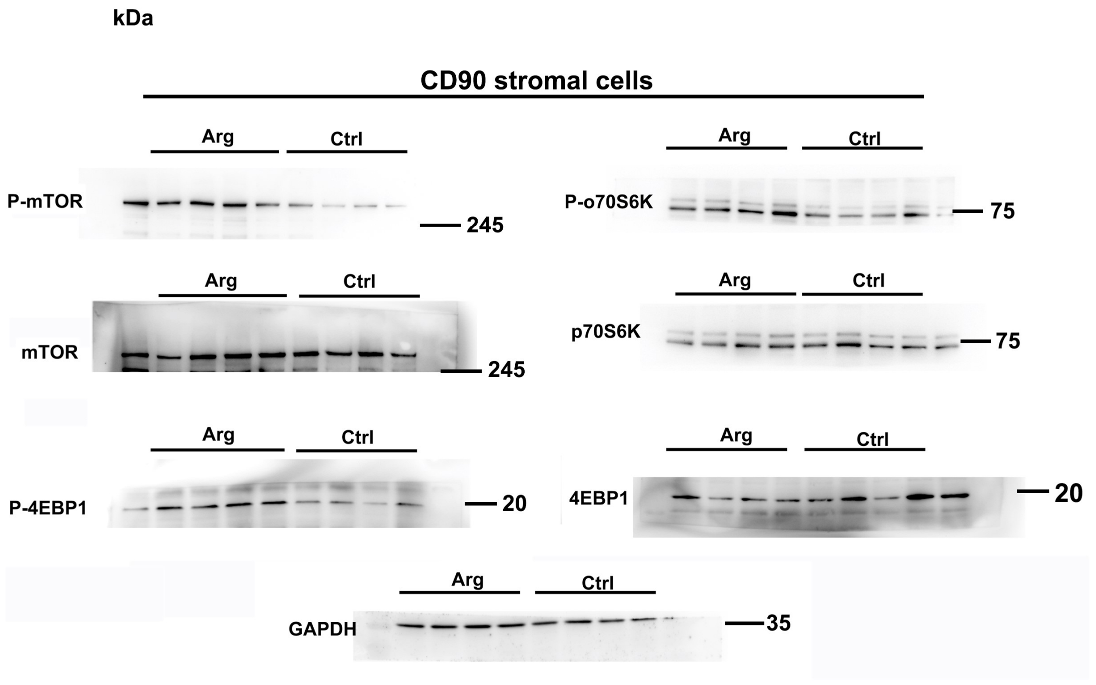

Original western blots in Figure 4B

kDa

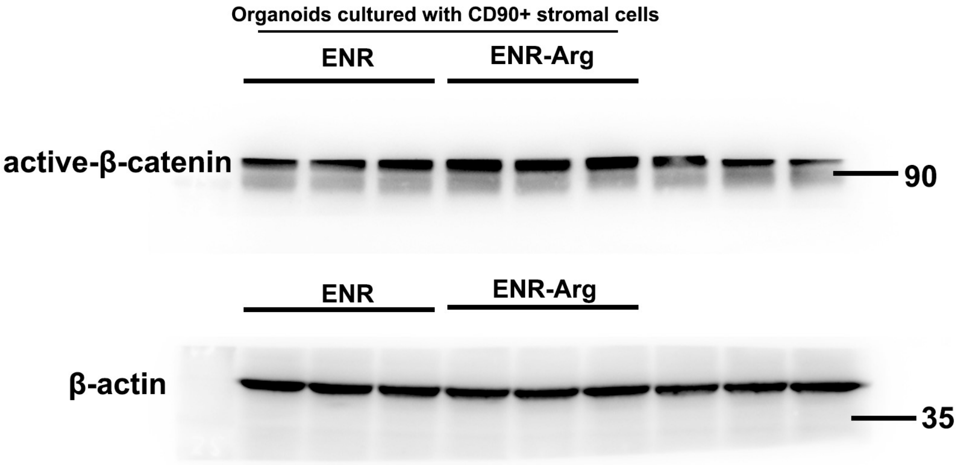

Original western blots in Supplementary Figure 3A

kDa

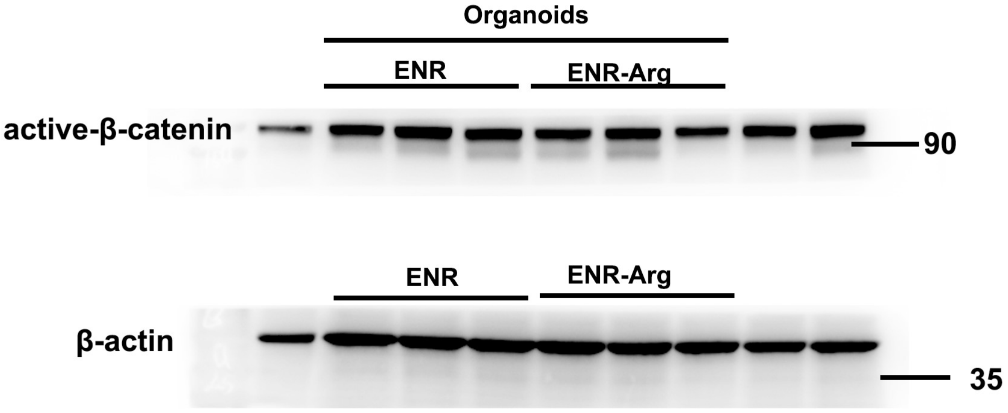

Supplement: Supplementary file 1 — Supplementary Information [file 42003_2020_1347_MOESM1_ESM.pdf]
