# Supplementary material for: Exogenous L-arginine increases intestinal stem cell function through CD90+ stromal cells producing mTORC1-induced Wnt2b
Source: Commun Biol. 2020 Oct 23;3:611. doi: 10.1038/s42003-020-01347-9 (PMC7584578; doi:10.1038/s42003-020-01347-9)
Supplement: Supplementary file 3 — Description of Additional Supplementary Items [file 42003_2020_1347_MOESM3_ESM.docx]

**Description of Additional Supplementary Files**

File Name: Supplementary Data 1

Description: Raw Data underlying the graphs.
